# Epidemiology and RAPD-PCR typing of thermophilic campylobacters from children under five years and chickens in Morogoro Municipality, Tanzania

**DOI:** 10.1186/s12879-016-2031-z

**Published:** 2016-11-21

**Authors:** Idrissa S. Chuma, Hezron E. Nonga, Robinson H. Mdegela, Rudovick. R. Kazwala

**Affiliations:** 1 Tanzania National Parks (Serengeti), P. O. Box 3134, Arusha, Tanzania; 2 Department of Veterinary Medicine and Public Health, Sokoine University of Agriculture, College of Veterinary and Medical Sciences, P. O. Box 3021, Morogoro, Tanzania

**Keywords:** *Campylobacter jejuni*, *C. coli*, *Epidemiology*, RAPD PCR, Genetic diversity, Children, Chicken, Morogoro, Tanzania

## Abstract

**Background:**

Campylobacter species are gram negative and flagellated bacteria under the genus *Campylobacter*, family Campylobacteriaceae. These pathogens cause zoonotic infections among human and animal populations. This study was undertaken between December 2006 and May 2007 to determine prevalence, risk factors and genetic diversity of thermophilic *Campylobacter* isolates from children less than 5 years and chickens in Morogoro Municipality, Tanzania.

**Methods:**

The Skirrow’s protocol was used for isolation and identification of *Campylobacter* from 268 human stool specimens and 419 chicken cloacal swabs. Patient biodata and risk factors associated with human infection were also collected. Genetic diversity of *Campylobacter* isolates was determined by a RAPD-PCR technique using OPA 11 primer (5′-CAA TCG CCG T-3′). Phylogenetic analysis and band pattern comparison were done by Bionumerics software and visual inspection.

**Results:**

Stool samples from 268 children and 419 cloacal swabs from chickens were analyzed. Prevalence of thermophilic *Campylobacters* in children was 19% with higher isolation frequency (*p = 0.046*) in males (23.5%) than females (13.8%). *Campylobacter jejuni* (78.4%) was more isolated (*p = 0.000*) than *C. coli* (19.6%) and 2% were unidentified isolates. In chickens, the prevalence was 42.5% with higher isolation rate (*p = 0.000*) of *C. jejuni* (87%) than *C. coli* (13%). *Campylobacters* were more frequently recovered (*p = 0.000*) from indigenous/ local chickens (75.0%) followed by cockerels (52.2%), broilers (50.0%) and lowest in layers (22.7%). Keeping chickens without other domestic animals concurrently (*p = 0.000*), chicken types (*p = 0.000*) and flock size (*p = 0.007*) were risk factors for infection in chickens. One hundred and fifty two (152) thermophillic *Campylobacter* isolates were genotyped by RAPD-PCR of which 114 were *C. jejuni* (74 from chickens and 40 humans) and 38 *C. coli* (28 from chickens and 10 humans). Comparison of *Campylobacter* isolates from children and chickens revealed high diversity with only 6.1% of *C. jejuni* and 5.3% of *C. coli* being 100% genetically similar.

**Conclusions:**

This study has recorded high prevalence of thermophilic *Campylobacter* in children less than 5 years and chickens in Morogoro municipality. The observed genetic similarity among few *C. jejuni* and *C. coli* isolates from children and chicken suggests existence of cross transmission of these pathogens between children under 5 years and chickens.

## Background

Thermophilic *Campylobacters* include *Campylobacter jejuni*, *C. coli* and *C. lari* have been causing gastroenteritis, which is a major public health concern worldwide since 1970s [1]. Ecologically, *Campylobacter* species are ubiquitous and may be isolated from gastrointestinal tract of reservoir animals in particular chicken and other birds [2]. In Tanzania, urban and peri-urban agriculture (UPA) has been expanding with chicken, cattle and pigs being major livestock species kept for meat production; dogs and cats for watching, controlling pests, hunting and as pets. Increasing urbanization with animals living in closer proximity with humans is among risks for transmission of zoonotic diseases including campylobacteriosis. Spread of zoonotic diseases may be enhanced by poor hygiene and sanitation; malnutrition; poor health status; poor feeding habits; poor immunity and HIV and AIDS [3–8]. Thermophilic *Campylobacter* isolation rates of up to 70% have been reported in chickens [9], ducks 80% [10], beef 9.3% [11], pork 10.6% [12] and milk 13.4% (Kashoma, 2015; personal communication). Consumption of raw or undercooked meat, raw milk and untreated water are among predisposing factors to *Campylobacter* infection in humans [3, 13–16]. These malpractices are not uncommon among people of diverse socio-cultural origins in rural settings of Tanzania. Limited water supply, lack of water treatment and contamination of water with pathogens [17–19] aggravates situation by increasing risks for *Campylobacter* infection both in human and animal populations.

Several epidemiological studies aimed at isolating thermophilic *Campylobacter* species, establish their prevalence and risk factors in Tanzania have been conducted in humans, poultry, cattle and water [3, 20, 21]. However, limited studies aimed at establishing the link between infections in humans and animals in Tanzania [3]. The current study was undertaken to positively contribute in bridging this gap of knowledge by exploring genetic relatedness among thermophilic *Campylobacter* isolates from human and chicken populations in Tanzania. Thus, this study reports prevalence, risk factors and genetic diversity of thermophilic *Campylobacter* isolates from children less than five years of age and chickens in Morogoro municipality, Tanzania. A simple, fast and cost effective Random amplified polymorphic DNA (RAPD) PCR assay [22] was employed to determine the genetic diversity of thermophilic *Campylobacter* isolates in this study. This study has enabled us to deduce existence of zoonotic transmissions of these bacteria between children less than 5 years and chicken populations in Morogoro, Tanzania.

## Methods

### Study area and sampling

This cross-sectional study was conducted in Morogoro Municipality, Tanzania (37°4'E; 4°49'S and altitude of 487–600 m above sea level) between December 2006 and May 2007. Aim of the current study was to determine the prevalence, risk factors for infection and genetic relatedness among thermophilic *Campylobacter* isolates from children below 5 years of age and chickens. Sample sizes were calculated using the formula *n = Z* ^*2*^ *p (1-p)/ d* ^*2*^ [23] where: *n* is sample size; *Z* is the multiplier from the normal distribution, *p* is the expected prevalence and *d* is the desired absolute precision. The expected prevalence of campylobacter infection (*p*) used for sample size estimation was *p* = 20% for humans [24] and *p* = 70% for chickens [25]. Other values (*Z, d,* and CI) were kept constant. With *Z* value of 1.96 at 95% confidence interval (CI) and desired precision (*d*) of 0.05, the calculated minimum sample sizes (*n*) were 250 and 330 for humans and chickens, respectively.

Children under 5 years of age attending Outpatient Department (OPD) at Morogoro Regional and Mazimbu Hospitals, Mafiga, Madizini, Usangi and Upendo health facilities were enrolled in this study. Children that were admitted, hospitalized and those under antibiotic therapy were excluded to avoid confounding effects on the bacterial isolation. Human stool samples were collected in clean sterile 10 ml-plastic containers by parents/guardians and submitted to the medical laboratory technicians. The samples were aseptically transferred into sterile 10 ml-universal bottles containing 5 ml of *Campylobacter* enrichment broth and stored at 4 °C before and during shipment to the laboratory. Biodata and the possible risk factors associated with human infection (age, sex, keeping chickens, keeping other animals and boiling or treating drinking water) were recorded. History of the study children experiencing a gastrointestinal disorder characterized by passing out loose and watery stool at least three times a day in the past 2 weeks and consistency of the stool samples were the criteria used to categorize the patients as diarrhoeic or non-diarrhoeic.

Chicken cloacal swab samples were collected from 22 chicken flocks/farms located in various areas within Morogoro Municipality. After collection, the swabs were put into universal bottles containing 5 ml of *Campylobacter* Enrichment Broth (Oxoid Ltd, Basingstoke, Hampshire, England). These samples were placed on ice blocks in a cool box at approximate temperature of 4 °C and transported to the laboratory within 2 h. Study chicken populations included indigenous/local chickens, broilers, layers and cockerels. Categories of chickens sampled are in line with types preferably kept by majority of farmers/livestock keepers in Morogoro municipality, Tanzania. These were: indigenous/local chickens are of mixed sexes and free ranging while broilers are chickens of mixed sexes kept for meat production. On the other hand, layers are all females kept for egg production and cockerels are all males kept for dual purposes namely meat and reproduction. The chickens were samples from 22 different flocks/farms located in various areas within Morogoro municipality. Among the indigenous/local chickens were those at the Morogoro Central Market ready for sale. For convenience, the flocks were classified as small when number of chickens of the chickens was categorized as 1 to 199, medium (200–299) and large (300 and above up to 7000). Age groups of the chickens were assigned as 0–4, 5–9, 10–14, 15–19 and 20 weeks and above (20+). It is worthwhile to note that age of 49 chickens could not be ascertained due to lack of proper record keeping and these were excluded in age related analysis. In addition, chickens less than 3 weeks of age and those under treatment with antibiotics were excluded to avoid confounding effects of the maternal immunity and negative growth of *Campylobacter* on the media, respectively. For convenience, flocks with 199 chickens and less were classified as small, those with 200–299 chickens as medium and flocks/farms with 300 and/ more as large. Information on chicken types, flock size, age and keeping of other animals was obtained by field observations and confirmed in interviews with the owners.

### Bacterial isolation and identification

Campylobacter species are relatively slow-growing, fastidious bacteria that require specialized culture conditions; hence, they grow best under reduced oxygen tension on nutritional basal media supplemented with 5–10% blood. In the laboratory, human stool samples and chicken cloacal swabs were aseptically inoculated in 10 ml-universal bottles containing 5 ml of Campylobacter Enrichment Broth (Lab M, International Diagnostics Group, plc, Lancashire, UK). The bottles were incubated at 37 °C for 24 h in an incubator (Heraeus B5050, Germany). Thereafter, one loopful of the enriched human or chicken samples was plated onto modified cefoperazone charcoal deoxychocolate agar (mCCDA) (Oxoid Ltd, Basingstoke, Hampshire, England) supplemented with CCDA selective supplement (Oxoid Ltd, Basingstoke, Hampshire, England). The plates were put in an anaerobic jar (Coldstream Engeneering Ltd, 18–10, Arista, Sweden) with microaerophilic environments generated by a lighted candle and then in the incubator (Memmert, Germany) at 43 °C for 48 h. Bacterial colonies suspected to be thermophilic campylobacter species based on growth at 43 °C and colony morphology were subjected to further examination by microscopy using Gram’s staining, motility and biochemical tests using Skirrow’s protocol as previously described [3]. Confirmed thermophilic Campylobacter isolates were sub-cultured on mCCDA (Oxoid Ltd, Basingstoke, Hampshire, England) and three loopfuls of 48-h old colonies were harvested and transferred into cryogenic vials (Nalgene®, Nalge Nunc Int. Corp, USA) containing 1 ml of brain heart infusion broth (Oxoid Ltd, Basingstoke, Hampshire, England) with 20–30% glycerol (v/v). The vials were incubated at 37 °C for 24 h, initially stored at -20 °C for 24 h and then transferred to -80 °C until when further analysis by RAPD-PCR genotyping was performed.

### RAPD-PCR genotyping

A total of 152 *Campylobacter* isolates were genotyped by RAPD-PCR using OPA 11 primer (5′-CAA TCG CCG T-3′) as described by Miwa et al. [7] and their genetic relatedness compared. Of these, 74 *C. jejuni* and 28 *C. coli* were isolated from chickens and 50 (40 *C. jejuni* and 10 *C. coli)* from humans. The DNA templates were prepared as described by Miwa et al. [7]. The RAPD reaction mixture consisted of 50 mM KCl, 10 mM Tris-HCl (pH 8.4 at 25 °C), 2.5 mM MgCl_2_, 0.1% Triton X-100, a 200 μM concentration of each deoxynucleoside triphosphate, 0.3 μM of the primer, 2.5U of *Taq* DNA polymerase (Invitrogen), 2.5 μl of the template DNA, and sterile nuclease-free water to a final volume of 25 μl. The 25 μl of reaction mixture was cycled in a Mastercycler (Eppendorf®, Germany) through the following temperature profile: an initial denaturation step at 94 °C for 1 min; 45 cycles of 94 °C for 1 min, 36 °C for 1 min, and 72 °C for 2 min; and a final elongation step at 72 °C for 5 min. The PCR products were held at 4 °C until when electrophoresis was performed.

### Gel electrophoresis and interpretation

Five microliter of amplified DNA fragments were electrophoresed alongside 3 μl of 1-kb ladder (Promega, Madison, USA) through 1% (w/v) agarose gels (Molecular grade - low EEO, Whitehead Scientific (Pty) Ltd) in 1X TBE buffer (0.45 M Tris, 0.44 M Boric acid and 0.01 M EDTA) (SIGMA®, Sigma Chemical Co., St Louis, USA). The agarose gels were electrophoresed at 60 V for 90 min, stained with ethidium bromide (Promega, Madison, USA) 0.005% (v/v) and photographed using a computerized image capturing machine, Kodak 4000®.

### Statistical analysis

Data were stored in a Microsoft Office Access database and analyzed using Epi-Info software [26]. Comparison of dichotomous variables was done using Chi-square (*χ* ^2^) test at a critical probability of 0.05 and 95% confidence interval. Gel images were imported to BioNumerics version 4.61 computer software (Applied Maths) for analysis and dendrogram production. Pairwise comparisons were accomplished using the Dice similarity coefficient, and the dendrograms were created using the unweighted pair group method using a geometric average (UPGMA). For the whole dataset, the most appropriate optimization and position tolerance settings, as determined by the software, were 0 and 1%, respectively. As the gel images could not be normalized, visual inspection was done to determine similarity of the RAPD profiles and their proportions expressed as percentage of the total number of *C. jejuni* and *C. coli* isolates analyzed.

## Results

### Thermophilic *Campylobacter* infection in children

Table 1 summarizes results for thermophilic campylobacter infection in children less than 5 years in Morogoro municipality, Tanzania. A total of 268 stool samples from children less than 5 years were analyzed of which 54% (n = 145) were males and 46% (n = 123) females. Prevalence of thermophilic *Campylobacter* infection was 19.0% with higher isolation frequency (*p = 0.000*) of *Campylobacter jejuni* (78.4%) than *C. coli* (19.6%), *C. lari* was not found and about 2% (n = 1) could not be identified. Higher prevalence (*p = 0.046*) was observed in males (23.5%) than females (13.8%).

**Table 1:** Risk factors and isolation frequency of thermophilic campylobacters in children <5 years in Morogoro

Risk factor	Group	Number of children/ chickens	Number of cases	Isolation frequency (%)	*P*-value	Remarks
Age (months)	3–13	51	7	13.7	*p* = 0.953	NS
	13–24	89	19	21.4		
	25–36	54	12	22.2		
	37–48	41	8	19.5		
	49–60	33	5	15.2		
Sex	Female	123	17	13.8	*p* = 0.046	S
	Male	145	34	23.5		
Keeping chickens	At home	84	10	11.9	*p* = 0.129	NS
	At neighbourhood	58	14	24.1		
	Not at all	126	27	21.4		
Keeping other animals	Kept	88	15	17.1	*p* = 0.496	NS
	Not kept	180	37	20.5		
Boiling/treating drinking water	Boiled/treated	161	27	16.7	*p* = 0.249	NS
	Unboiled/untreated	107	24	22.4		
Diarrhoea	Diarrhoeic	78	10	12.8	*p* = 0.066	NS
	Non-diarrhoeic	190	41	21.6		
Thermophilic Campylobacter isolates	*C. jejuni*	51	40	78.4	*p* = 0.000	S
	*C. coli*	51	10	19.6		
	*C. lari*	51	0	0.0		
	Unidentified	51	1	2.0		
	Overall	268	51	19.0		

S = statistically significant (*p* < 0.05) while NS = statistically not significant (*p* > 0.05)

**Fig. 1:**
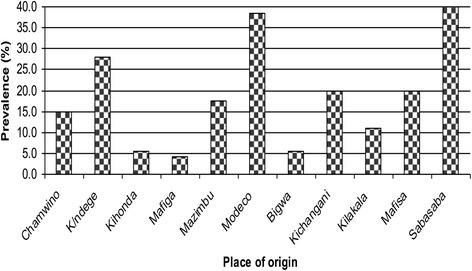
Isolation frequency and origin of children infected with thermophilic campylobacters in Morogoro

Prevalence of *Campylobacter* infection in non-diarrhoeic children (21.6%) and diarrhoeic (12.8%) was comparable (*p = 0.098*). Fig. 1 shows higher frequency of isolation (*p = 0.048*) of thermophilic *Campylobacters* in children originating from densely populated localities namely Sabasaba (40.0%), Modecco (38.5%) and Kiwanja cha ndege (K/ndege) (28.0%) while their counterparts from less populated areas including Bigwa, Kihonda, Kilakala and Mafiga (which is an exception in this case) had relatively lower frequencies (<12.0% each).

### Thermophilic *Campylobacter* infection in chickens

A total of 419 chicken cloacal swabs were collected from 22 chicken flocks located in different areas within Morogoro Municipality. Thirty seven percent (37%) of samples were obtained from broilers, 42.0% layers, 15.3% indigenous and 5.5% cockerels. Prevalence of thermophilic campylobacter infection in chickens was 42.5% (Table 2).

**Table 2:** Risk factors and isolation frequency of thermophilic campylobacters in chickens in Morogoro

Risk factor	Group	Number of children/chickens	Number of cases	Isolation frequency (%)	*P*-value	Remarks
Chicken types	Broilers	156	78	50.0	*p* = 0.000	S
	Cockerels	23	12	52.2		
	Indigenous	64	48	75.0		
	Layers	176	40	22.7		
Flock size	Small	199	75	37.7	*P* = 0.007	S
	Medium	19	0	0.0		
	Large	201	103	51.2		
Keeping other animals concurrently	Kept	242	76	31.4	*P* = 0.000	S
	Not kept	177	102	57.6		
Age^a^ (weeks)	0–4	11	3	27.3	*P* = 0.179	NS
	5–9	48	7	14.6		
	10–14	28	6	21.4		
	15–19	93	62	66.7		
	20+	190	56	29.5		
Thermophilic Campylobacter isolates	*C. jejuni*	178	155	87.1	*p* = 0.000	S
	*C. coli*	178	23	12.9		
	*C. lari*	178	0	0.0		
	Overall	419	178	42.5		

S = statistically significant (*p* < 0.05) while NS = statistically not significant (*p* > 0.05)

^a^49 indigenous chickens were excluded as their age could not be ascertained due to lack of proper records

Comparison of proportions of the isolated species (*p = 0.000*) revealed that *C. jejuni* was the most predominant species (87.1%) followed by *C. coli* (12.9%) and *C. lari* was, again, not found. Higher isolation frequency (*p = 0.000*) was observed in indigenous chickens (75.0%) than cockerels (52.2%), broilers (50.0%) and layers (22.7%). Risk factors for *Campylobacter* infection showed that larger and medium sized flocks had higher isolation frequencies of 51.2% and 37.7% respectively as compared to medium flocks that had no isolates (*p = 0.007*). Other risks (*p = 0.000*) were keeping: chickens alone (57.6%) not concurrently with other domestic animals (31.4%), indigenous/ local chickens (75.0%) than cockerels (52.2%), broilers (50.0%) and layers (22.7%).

### Genetic diversity of thermophilic *Campylobacter* isolates

**Fig. 2:**
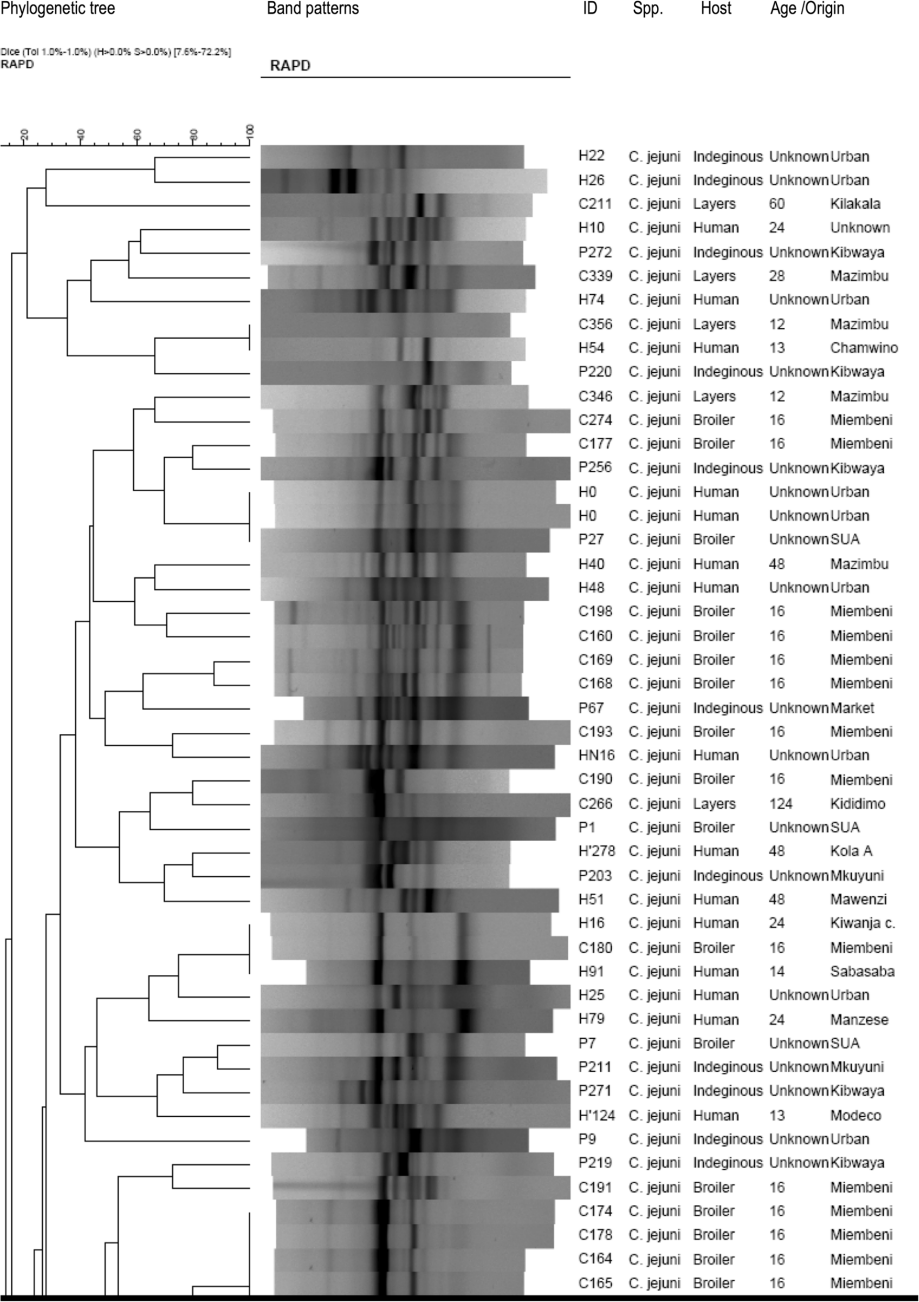
Part of phylogenetic tree showing genetic diversity among *C. jejuni* isolates from children and chickens in Morogoro

**Fig. 3:**
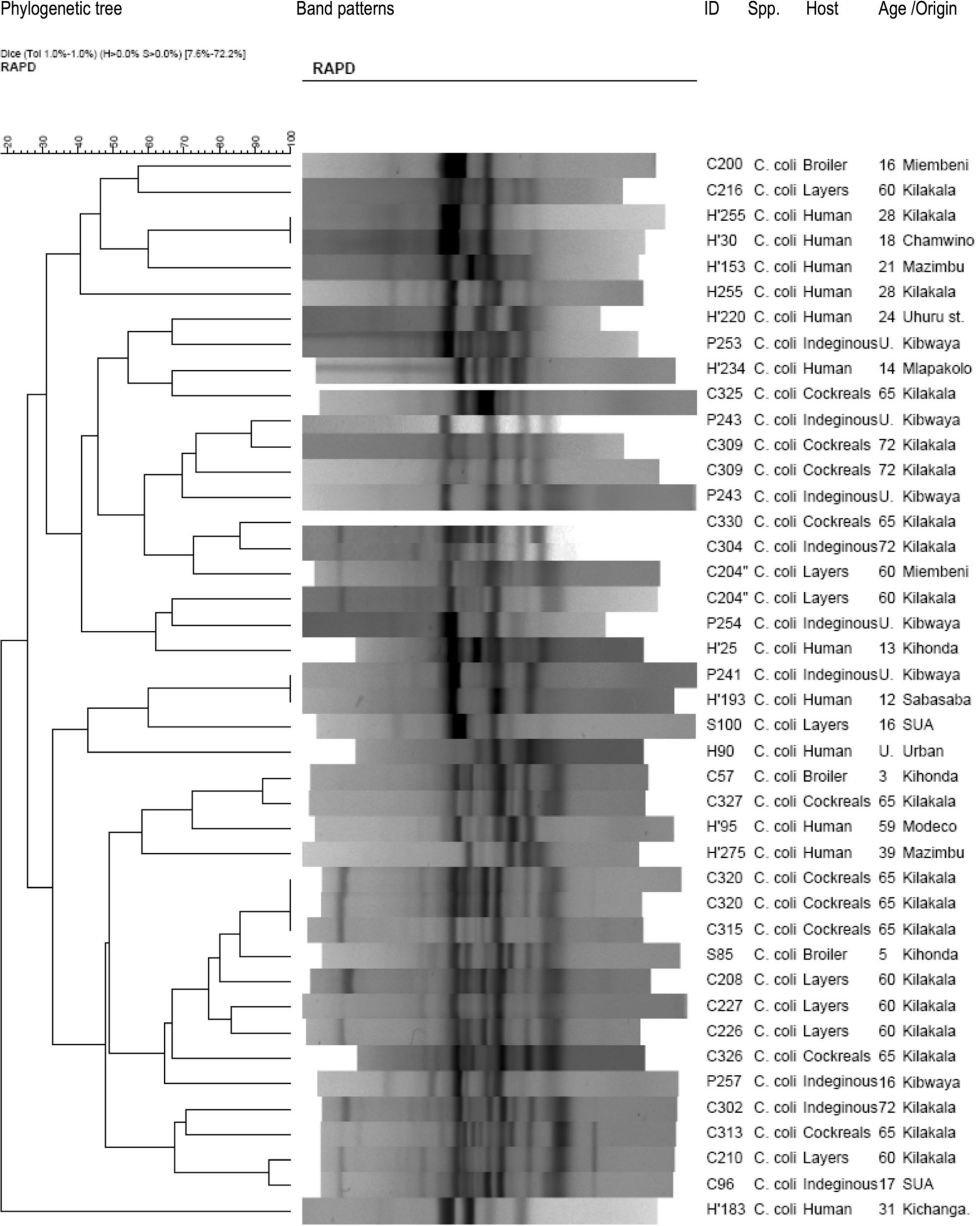
Phylogenetic tree showing genetic diversity among *C. coli* isolates from children and chickens in Morogoro

Of all 152 *Campylobacter* isolates genotyped by RAPD-PCR, 114 were *C. jejuni* (74 from chickens and 40 humans) and 38 *C. coli* (28 from chickens and 10 humans). All the isolates successfully generated bands whose patterns were analyzed (Figs. 2 and 3); however, P29 yielded no bands and was not included in the analysis. *C. jejuni* isolates were grouped into 28 clusters at 85% homology each with one to seven individuals. The clusters could not be linked to the geographical origins of the samples. *C. jejuni* and *C. coli* from both children and chickens were genetically highly diverse and not host-specific. Seven *C. jejuni* isolates (6.1%) and two *C. coli* isolates (5.3%) from the children and chickens had comparatively similar band patterns. The isolates and their origins in brackets were: C156 (Mazimbu) and H54 (Chamwino); H0 (Urban) and P27 (SUA); H16 (Kiwanja cha ndege), H91 (Sabasaba) and C180 (Miembeni) for *C. jejuni* and P241 from Kibwaya and H’193 from Sabasaba for *C. coli.* This finding implies probable sharing of these pathogens between humans and chickens. RAPD PCR was sensitive enough to identify some *C. jejuni* (C174, C178, C164, C165) and *C. coli* (C320 and 315) isolates from same flocks with 100% similarity. Four human *C. jejuni* isolates (H13, H91, H255 and H30) and two *C. coli* isolates H255 and H30) from different geographical locations were also 100% similar. It is worthwhile to note that five out of 114 *C. jejuni* (P216, P104, H0, H21 and H14) and four of 38 *C. coli* (P243, C309, C320, and C204) were run in duplicates (pairs) and they generated the same band patterns. Eight *C. jejuni* and *C. coli* isolates (7% of all 114 isolates) that came from different geographical origins were 100% similar. The similarity was observed among four *C. jejuni* and four *C. coli* (C191 and H234; C183 and S100; C163, C186, H255 and H30).

## Discussion

Diseases characterized by diarrhoea cause high mortalities in children under the age of five, old people above 65 years and immunocompromised individuals in Tanzania [27]. Children below 5 years and the young are more affected by *Campylobacter* enteritis than adults (3, 22]. Preponderance of infection in younger children could be due to poor hygiene and sanitation, malnutrition, intimacy to animals, health status, feeding habits and poor immunity [3–6, 8, 28, 29]. In other studies, keeping animals in close proximity with humans was a risk factor for children infection. For example, it was previously reported [8] that children from families that kept poultry and cattle had significantly higher prevalence of *Campylobacter* infections (27.2%) than their counterparts without animals (3.4%). Previous studies also demonstrated that toddlers in families keeping chickens had an average of 4 faeces-to-mouth episodes in 12-h [24]. The fact that poultry faeces can yield viable *C. jejuni* for at least 48 h after deposition suggests existence of a high risk of *Campylobacter* transmission in environments where there may be frequent human-animal contacts [29]. In the current study, keeping of chickens or other animals had no influence on infection of children. Therefore, this could be concluded that presence of animals coupled with poor hygiene and interaction between humans and animals is the risk factor for *Campylobacter* infection rather than a mere keeping of animals.

The prevalence of *Campylobacter* infection in children observed in this study (19%) is comparable to 18% reported in children less than 5 years in Dar es Salaam, Tanzania [30]. Comparable prevalence of *Campylobacter* infections in children less than 5 years reported elsewhere in the world include: 15.3% in West Bengal [8], 16.5% in Nigeria [31], 17.4% in Bangladesh [32] and 18% in Lawalpindi and Islamabad [28]. Higher prevalence of *C. jejuni* than *C. coli* coupled with low or lack of *C. lari* isolation was consistently reported in different countries [1, 3, 5, 9]. This may be due to differences in mechanisms of pathogenesis between *C. jejuni* and *C. coli* within the host cells [33]. *Campylobacter coli* are more readily phagocytosed and killed by peritoneal macrophages than *C. jejuni* [33]. This may also happen *in situ* in human cells and may explain the lower severity of infections due to *C. coli* as compared to *C. jejuni.* For example, *C. coli* were less often associated with bloody diarrhea and symptomatic disease as compared to *C. jejuni* [34]. However, variations in isolation frequencies of microorganisms may reflect actual differences in composition of common *Campylobacter* species in local environments.

Higher infection rate observed in male compared to female children supports findings by other studies that reported male to female risk ratios of 2:1 [31] and 1.7:1 [28]. However, other studies elsewhere found no difference in terms of sex of the infected people [3, 5]. In Tanzania, the actual cause of higher prevalence in males than females is yet to be established and further studies are recommended to elucidate sex predisposition to *Campylobacter* infection.

Higher prevalence of *C. jejuni* in chicken than *C. coli* and lack of *C. lari* isolation are in agreement with other previous findings [3, 36, 37]. *Campylobacter jejuni* is reported to behave as a commensal to a wide range of warm-blooded animals including avian hosts despite its being pathogenic to humans [37, 38]. The prevalence of thermophilic *Campylobacter* infection of 42.5% in chickens revealed by the current study is comparable to 47.8% [8] but by far lower than 70% reported earlier [25]. Variations in isolation rates may be explained by the actual differences in local prevalence of *Campylobacter* in a specific region, seasonality, chicken management system, sampling techniques and laboratory methodologies employed. For example, sampling of chicken faeces by using cloacal swabs was found to be less sensitive as compared to the intestinal contents [25]. Similarly, Cape Town protocol was reported to be a superior technique over Skirrow’s method in isolation of *Campylobacter* [39]. In addition, it was established that at a certain stage of colonization process *Campylobacter* species can only be found in the caecum and they cannot not be shed in faeces [40]. At such a stage, caecum is the best colonization site and not elsewhere.

The current study reports higher prevalence of *Campylobacter* infection in indigenous local chickens than broilers, layers and cockerels (*P < 0.05*). A number of studies have highlighted the role of poultry, especially chickens in the transmission of *Campylobacter* to humans. Majority of chickens in Tanzania are indigenous and reared under free-range system with no or minimal veterinary attention posing more public health concerns. These chickens do freely move and spread their droppings around homesteads and contaminated environments serve as potential sources of infection to children that prefer playing nearby.

*Campylobacter* loads are known to increase with age in broilers as previously reported [4, 25, 35] and levels of up to 1.2 × 10^7^c.f.u/g have been reported in poultry [36]. Four-week old chickens had prevalence of 64.3% but at the age of 39 weeks the levels significantly increased to 93%. *Campylobacter* are in most cases commensal to the chicken gastrointestinal track, older chickens are likely to have more *Campylobacter* load because of prolonged exposure. This is caused by interactions with various sources of infection with larger numbers and diverse strains that they come across with as they age predisposing the chickens to infections. In the current study, thermophilic *Campylobacter* species were isolated from chickens of all age groups and insignificantly higher isolation frequency was observed in chickens aged 15–19 weeks as compared to other age.

The observed highly diverse band patterns imply existence of genetically distinct individuals or strains of *C. jejuni* and *C. coli* in human and chicken populations in Morogoro Municipality. Genetic diversity is one of several mechanisms that enable pathogens thrive in adverse conditions within the host and/or in the environments giving them ability to colonize multiple animal hosts. This phenomenon is important in perpetuating *Campylobacter* infections in human and animal populations. Genomic rearrangement and horizontal gene transfer are among various mechanisms that can generate genomic diversity. These adaptive mechanisms could have contributed to the currently observed thermophilic *Campylobacter* diversity [41]. Alterations in genetic make up of microorganisms could also be due to mutations, which may occur spontaneously in normal biological life.

A small proportion of 100% similar band patterns among *C. jejuni* (6.1%) and *C. coli* isolates (5.3%) from humans and chickens was observed implying that the *Campylobacter* were shared between humans and chickens in Morogoro Municipality. Previous studies in different parts of the world reported similarity among thermophilic *Campylobacter* isolates from human and chickens [2, 42, 43]. In Poland, cluster analysis of RAPD patterns from 115 *Campylobacter* isolates from chickens and 80 from children revealed that six human *C. coli* isolates were identical to chicken isolates [43]. In Barbados, the OPA 11 fingerprinting of one *C. jejuni* and five *C. coli* isolates from human were identical to one *C. jejuni* and five *C. coli* from chicken meat, respectively [2]. In Canada, macrorestriction profiles of approximately 20% of human *Campylobacter* isolates were genetically related to genotypes found in poultry [42].

## Conclusions

Thermophilic *Campylobacter* infection is prevalent both in children less than 5 years of age and chickens in Morogoro Municipality, Tanzania. *Campylobacter jejuni* is the leading cause of *Campylobacter* infections in children and chicken followed by *C. coli* while *C. lari* was not isolated*.* Both *C. jejuni* and *C. coli* isolates from humans and chickens showed high genetic diversity with only 6% of *C. jejuni* and 5% of *C. coli* from the two hosts being genetically similar (100%). These thermophilic bacteria were most likely shared between children and chicken populations under this study in Morogoro Municipality. More genetic and epidemiological studies are recommended in Tanzania and elsewhere in the world to further explore genetic relatedness among thermophilic *Campylobacter* isolates from humans and both domestic and wild animals especially those at a close proximity to humans in day-to-day life in Tanzania.

## Abbreviations

BTC: Belgian Technical Cooperation
NIMR: National Institute for Medical Research in Tanzania
NVH: Norges veterinærhøgskole (Norwegian School of Veteinary Science)
OPD: Outpatient Department
PANTIL: Programme for Agriculture and Natural Resource Transformation for Improved Livelihood
PCR: Polymerase chain reaction
RAPD: Random amplified polymorphic DNA
SUA: Sokoine University of Agriculture
TANAPA: Tanzania National Parks
